# Spatial and Temporal Characteristics of Phytoplankton Communities in Drinking Water Source Reservoirs in Shenzhen, China

**DOI:** 10.3390/plants12233933

**Published:** 2023-11-22

**Authors:** Qiting Liang, Xingliang Jin, Jie Feng, Shenhao Wu, Jiajia Wu, Ying Liu, Zixin Xie, Zhi Li, Chunxing Chen

**Affiliations:** 1 Shenzhen Academy of Environmental Sciences, Shenzhen 518000, China; 15626471857@163.com (Q.L.); wu.shenhao@outlook.com (S.W.); wu-jia-jia@outlook.com (J.W.); liuying@sz.pku.edu.cn (Y.L.); 15818536522@163.com (Z.X.); lz18677907@163.com (Z.L.); chencx@126.com (C.C.); 2 Shenzhen Ecological Environment Monitoring Station, Shenzhen 518000, China; xingliangjin@hotmail.com

**Keywords:** phytoplankton community, diversity, environmental factors, drinking water reservoirs, eutrophication

## Abstract

Phytoplankton diversity and community characteristics are closely associated with aquatic environmental factors. Understanding these dynamics can provide insights into the ecological health of water bodies. We investigate the spatial and temporal characteristics of phytoplankton communities in 27 drinking water source reservoirs in Shenzhen, China. As a method, we collected samples during the dry season in 2021 and the wet season in 2022, analyzed the alpha and beta diversities of phytoplankton communities, and correlated these with the environmental factors. The results reveal that Cyanobacteria dominate the phytoplankton communities in the Shenzhen reservoirs. Phytoplankton diversity is greater during the dry season. The algal composition varies spatially, and the phytoplankton diversity tends to decrease with increasing eutrophication. A co-occurrence network analysis indicates denser and stronger correlations among phytoplankton nodes during the wet season than dry season. Reservoirs with moderate eutrophication levels exhibit denser nodes and stronger correlations compared to those with low or high eutrophication levels. The chemical oxygen demand, water temperature, pH, and total nitrogen are identified as key influencers of the phytoplankton community structure. Our results contribute to the enhanced understanding of the spatial and temporal dynamics of phytoplankton communities in reservoirs in South China and provides insights into the management and conservation of these drinking water reservoirs.

## 1. Introduction

Reservoirs are artificial multi-functional aquatic ecosystems that provide various ecosystem services for social development (e.g., economic, regulation, and cultural values) and represent an important water resource guarantee for social sustainable development [1,2]. China has more reservoirs than any other country, with dams built on almost all major and minor rivers except for Nujiang and Yarlung Zangbo, totaling more than 98,000 [3]. With the growth of the population and the increasing demand for water, the functions of these reservoirs have changed. These reservoirs were originally only used for flood control or irrigation, and they are now also used for drinking. However, the water quality requirements for drinking water are higher than those for flood control and irrigation, and the increased population, food supply, land conversion, and fertilizer use may increase the eutrophication and cyanobacterial blooms in these reservoirs [4,5].

Phytoplankton communities are abundant and important biological groups in aquatic ecosystems, and they play crucial roles in the ecosystem structure and function. As primary producers in food webs, phytoplankton supplies oxygen and nutrients to herbivores, and microorganisms decompose organic matter, recycle nutrients, and maintain the ecosystem balance [2,6,7]. In developing countries, industrialization and urbanization have led to the deterioration of local freshwater quality, resulting in frequent occurrences of water body eutrophication. Effectively managing eutrophication in these waters is crucial for ensuring drinking water safety, protecting human health, and promoting sustainable economic and social development. The diversity and composition of phytoplankton communities are commonly utilized as reliable indicators of water quality due to their short life cycles and high sensitivity to environmental variations [8]. It is crucial to enhance our understanding of the phytoplankton composition in drinking water reservoirs and the environmental factors that influence the phytoplankton community at this stage.

The mechanisms of phytoplankton community succession in freshwater and marine environments have been extensively investigated. The seasonal dynamics of phytoplankton communities are determined largely by seasonal fluctuations in the environmental variables, nutrient levels, and biological interactions. Most studies have examined the associations between phytoplankton distribution and physicochemical properties. Many lakes are thermally stratified, and water mixing jointly affects light penetration and nutrient concentrations, which affect phytoplankton growth [9,10]. However, few studies have described general trends in phytoplankton diversity and dynamics in subtropical reservoirs, and the key drivers of phytoplankton succession remain poorly understood, especially in drinking water source reservoirs.

Most global reservoirs have experienced rapid rises in eutrophication, with water quality issues often linked to nutrient loading [11]. Although the pattern of eutrophication and cyanobacterial blooms has been reported along latitudinal climate gradients, spatial differences in the sensitivity of aquatic ecosystems can be considerable. Therefore, studies that assess the vulnerability of water bodies at specific locations are needed by local governments to support and devise appropriate management actions. Because water quality degradation associated with phytoplankton significantly adversely affects aquatic ecosystems, understanding the seasonal succession of phytoplankton and the main driving environmental factors is needed for effective reservoir drinking water quality management.

Since few studies have investigated phytoplankton communities and their diversity at both spatial and temporal scales, we examine phytoplankton diversity, symbiotic networks, and the correlation with environmental variables in 27 drinking water reservoirs in Shenzhen during the dry and wet seasons. We aim to describe (1) the characteristics of phytoplankton communities in drinking water reservoirs in Shenzhen; (2) the temporal and spatial variations in phytoplankton communities; (3) the main environmental factors affecting phytoplankton communities; and (4) the co-occurrence patterns among the phytoplankton members. This study will advance the understanding of aquatic ecosystems in drinking water reservoirs in Shenzhen and help improve the management policies of water quality in reservoirs in Southern China.

## 2. Results

### 2.1. Phytoplankton Community Composition

A total of 54 water samples were collected from 27 drinking water reservoirs in Shenzhen during the dry season in 2021 and the wet season in 2022. A total of 66 genera of phytoplankton attributed to seven units that were identified using classical morphology: Chlorophyta (32 genera, 48% of all species), Cyanobacteria (12 genera, 18% of all species), Bacillariophyceae (11 genera, 17% of all species), and Dinoflagellata, Euglenophyta, Chrysophyceae, and Cryptophyceae (4, 3, 2, and 2 genera, respectively, representing 6%, 5%, 3%, and 3% of all species, respectively) (Figure S1). Figure S2 presents Venn diagrams depicting characteristics of the community structure between seasons and groups. The numbers of genera in the dry and wet seasons were similar (60 and 58 genera, respectively). During the dry season, 25 genera were common to Groups A, B, and C, with Groups A and C having similar numbers of species; Group B had the lowest number of species. In the wet season, 25 genera were common to Groups A, B, and C, with Group A having the most species, and Group C having the least.

**Figure 1:**
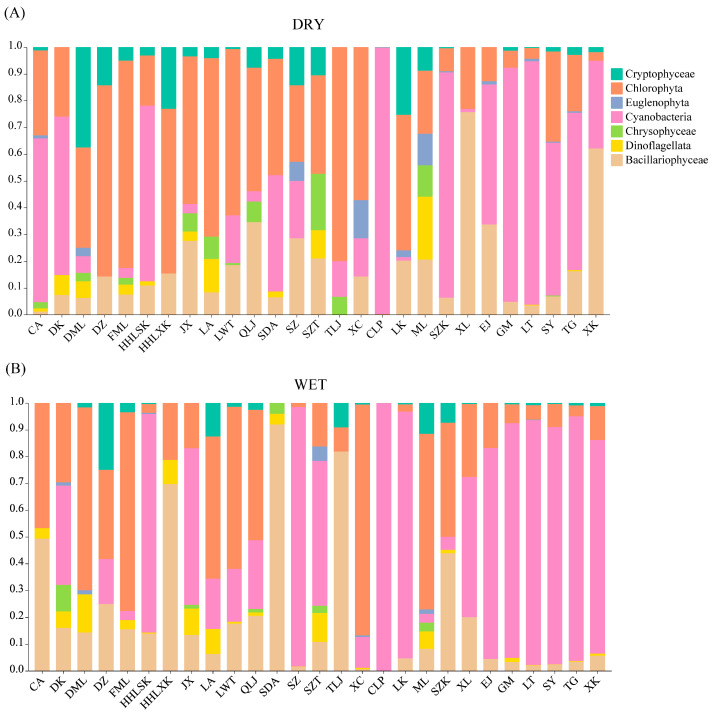
Algal composition of 27 drinking water reservoirs in Shenzhen. (**A**) dry season, (**B**) wet season. Dry and Wet represent dry and wet seasons, respectively.

During the dry season of 2021, the numerically dominant phytoplankton were Cyanobacteria (73%), Chlorophyta (12%), and Bacillariophyceae (13%). The wet-season-dominant phytoplankton were Cyanobacteria (92%) and Chlorophyta (5%) (Figure S1). Group A had greater Chlorophyta and lower Cyanobacteria abundances in the dry season, and Group C had a greater Cyanobacteria abundance; the Cyanobacteria abundances in Group B were between those of Groups A and C. Similar patterns occurred in these groups during the wet season (Figure 1). The dominant phytoplankton taxa are detailed in Table 1. The dry-season-dominant taxa are referrable to Cyanobacteria, Chlorophyta, and Bacillariophyceae, with a rich variety of dominant species (e.g., species of *Chlorella*, *Cryptomonas*, *Melosira*, *Microcystis*, *Pseudanabaena*, and *Rhizosolenia*). However, during the wet season, Cyanobacteria dominated in most reservoirs, with *Pseudanabaena* sp. Being dominant in the Group B and C reservoirs, and *Cyclotella* sp., *Coelastrum* sp., and some filamentous Cyanobacteria being dominant in the Group A reservoirs. Cyanobacteria (such as *Pseudoanabaena* sp. and *Microcystis* sp.) are dominant species in some reservoirs (such as DK, SZK, CLP, EJ, GM, SY, and TG) during both the dry and wet seasons.

### 2.2. Phytoplankton Community Alpha and Beta Diversities

**Figure 2:**
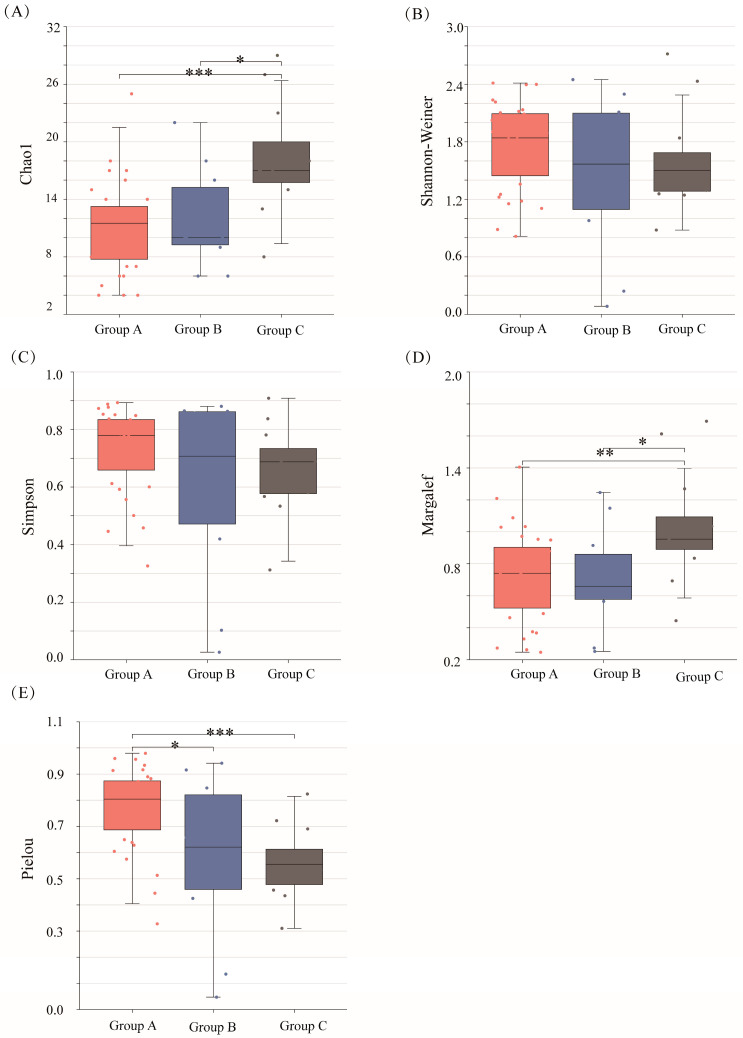
Alpha diversity results for 27 reservoirs. Indexes: (**A**) Chao, (**B**) Shannon−Weiner, (**C**) Simpson, (**D**) Margalef’s, (**E**) Pielou’s. *: *p*-value < 0.05; **: *p*-value < 0.01; ***: *p*-value < 0.0001.

We appraise the phytoplankton community alpha diversity using the Shannon–Wiener, Simpson, Chao1, Pielou’s, and Margalef’s diversity indexes (Figure 2 and Figure S3). The mean values of the Simpson and Shannon–Wiener indexes are higher during the dry season than the wet season. Conversely, the mean values of the Chao1, Pielou, and Margalef’s indexes are lower during the dry season than during the wet season. This indicates that during the dry season, species with low population numbers are relatively more abundant and have a higher species diversity, but their distributions are more uneven, causing reduced species richness and decreased density. Group A has the highest mean Simpson, Shannon–Wiener, and Pielou’s index values, and Group C has the lowest values. Conversely, Group C has the highest mean Chao1 and Margalef’s index values, and Group B has the lowest. Group A has higher relative abundances of less dense species, greater species diversity, and a more even distribution of species than Group B, but lower species richness and density. Group B has a higher relative abundance of less dense species and a higher and more even species diversity than Group C, but (similar to Group A) fewer species and lower species densities. While none of the diversity indexes differ significantly between the dry and wet seasons, significant differences are observed in the Chao1, Margalef’s, and Pielou’s indexes among the reservoir groups.

**Figure 3:**
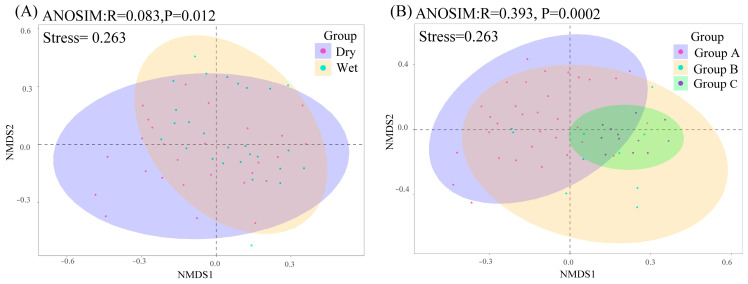
Nonmetric multidimensional scaling analysis (NMDS) and analysis of similarities (ANOSIM) of phytoplankton communities. (**A**) comparison between wet and dry seasons; (**B**) comparison between Groups A, B, and C. Dry and Wet represent dry and wet seasons, respectively.

While the alpha diversity in the Shenzhen drinking water reservoirs did not differ significantly over time, there were significant spatial differences. The NMDS plots and similarity analysis (ANOSIM) based on the Bray–Curtis distance revealed seasonal (R = 0.083, *p* = 0.012) and spatial (R = 0.383, *p* = 0.002) differences in the phytoplankton community composition, suggesting that a greater variability between the communities occurred in space than in time. Samples from specific seasons or groups were grouped together more closely (Figure 3). The SIMPER (similarity percentage) analyses revealed that *Pseudanabaena* sp., *Microcystis* sp., *Melosira* sp., *Cylindrospermopsis* sp., and *Coelastrum* sp. contributed the most to the spatial and temporal differences in the phytoplankton biomes (Supplemental Table S3).

### 2.3. Relationships between Phytoplankton Communities and Environmental Factors

**Figure 4:**
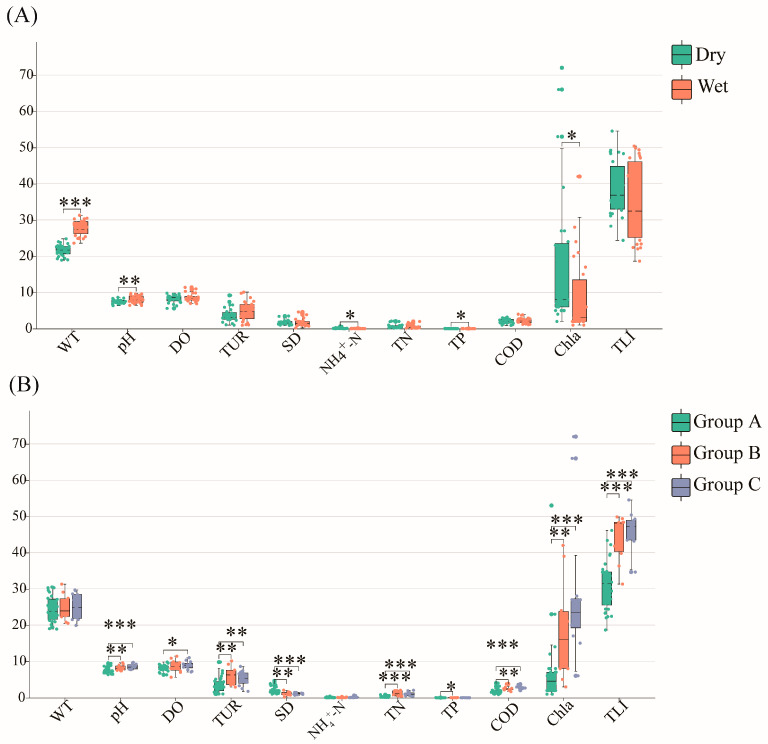
Box plot diagrams for water quality conditions. (**A**) Box plots of water quality conditions during dry and wet seasons. (**B**) Box plots of water quality for Groups A, B, and C. The meanings of the abbreviations are as follows: water temperature (WT), pondus hydrogenii (pH), dissolved oxygen (DO), turbidity (Tur), ammonia nitrogen (NH_4_^+^–N), total phosphorus (TP), chemical oxygen demand (COD), total nitrogen (TN), chlorophyll a (Cha), transparency (SD), and trophic level index (TLI). Dry and Wet represent dry and wet seasons, respectively. *: *p*-value < 0.05; **: *p*-value < 0.01; ***: *p*-value < 0.0001.

The relationships between the phytoplankton communities and 11 environmental variables, including the water temperature (WT), pondus hydrogenii (pH), dissolved oxygen (DO), turbidity (Tur), ammonia nitrogen (NH_4_^+^–N), total phosphorus (TP), chemical oxygen demand (COD), total nitrogen (TN), chlorophyll a (Cha), and transparency (SD), and the trophic level index (TLI) were examined. These variables differ significantly seasonally and spatially (Figure 4). The Mantel test reveals a strong correlation between the Tur and phytoplankton communities (r > 0.4, *p* < 0.01) during the dry season. Moreover, the WT, pH, DO, Tur, and COD were strongly correlated with the phytoplankton composition in multiple groups. The WT was significantly correlated in Groups B (r > 0.2, *p* < 0.05) and C (r > 0.2, *p* < 0.05); the pH is significantly correlated in Groups A (r ≥ 0.4, *p* < 0.01) and C (r > 0.2, *p* < 0.05); the DO is significantly correlated in Groups A (r > 0.2, *p* < 0.05) and B (r > 0.2, *p* < 0.05); and the Tur (r ≥ 0.4, *p* < 0.01) and COD (r > 0.2, *p* < 0.05) are significantly correlated with the phytoplankton communities in Group B.

**Figure 5:**
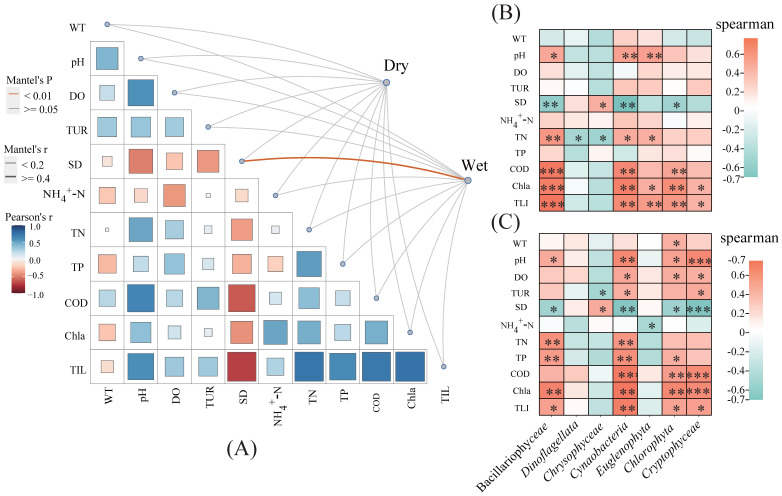
Environmental factors affecting phytoplankton communities. Environmental drivers of phytoplankton communities, as evaluated via Mantel tests in (**A**) wet and dry seasons. Correlations between major contributing taxa and environmental variables in (**B**) dry season and (**C**) wet season. The meanings of the abbreviations are as follows: water temperature (WT), pondus hydrogenii (pH), dissolved oxygen (DO), turbidity (Tur), ammonia nitrogen (NH_4_^+^–N), total phosphorus (TP), chemical oxygen demand (COD), total nitrogen (TN), chlorophyll a (Cha), transparency (SD), and trophic level index (TLI). Dry and Wet represent dry and wet seasons, respectively. *: *p*-value < 0.05; **: *p*-value < 0.01; ***: *p*-value < 0.0001.

**Figure 6:**
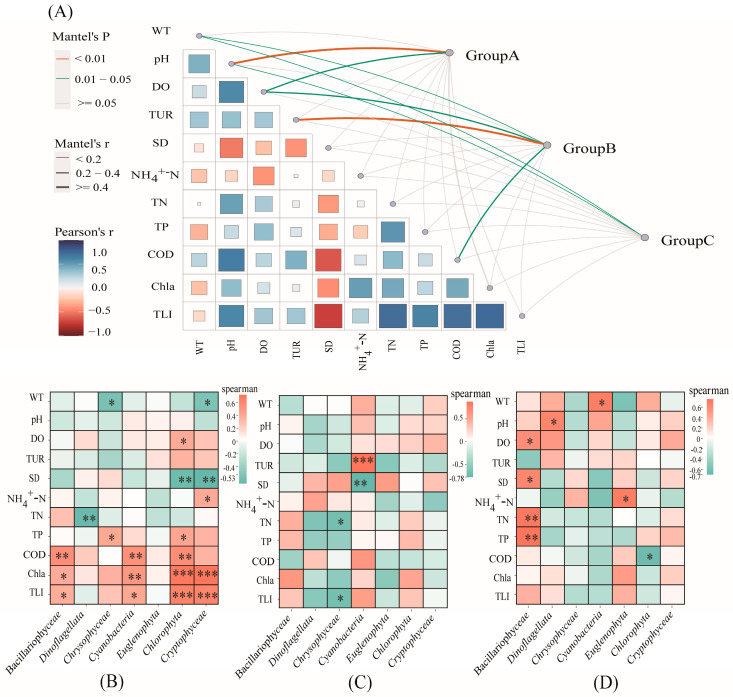
Environmental factors affecting phytoplankton communities. Environmental drivers of phytoplankton communities, as evaluated via Mantel tests in (**A**) Groups A, B, and C. Correlations between major contributing taxa and environmental variables in (**B**) Group A; (**C**) Group B; and (**D**) Group C. The meanings of the abbreviations are as follows: water temperature (WT), pondus hydrogenii (pH), dissolved oxygen (DO), turbidity (Tur), ammonia nitrogen (NH_4_^+^–N), total phosphorus (TP), chemical oxygen demand (COD), total nitrogen (TN), chlorophyll a (Cha), transparency (SD), and trophic level index (TLI). *: *p*-value < 0.05; **: *p*-value < 0.01; ***: *p*-value < 0.0001.

**Figure 7:**
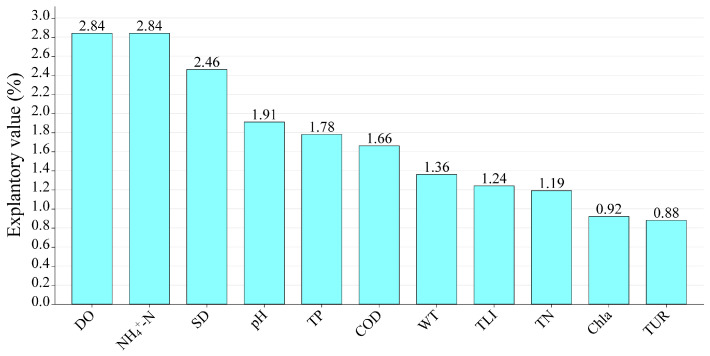
Variation partitioning analysis (VPA) of effects of environmental factors on community structure. The meanings of the abbreviations are as follows: water temperature (WT), pondus hydrogenii (pH), dissolved oxygen (DO), turbidity (Tur), ammonia nitrogen (NH_4_^+^–N), total phosphorus (TP), chemical oxygen demand (COD), total nitrogen (TN), chlorophyll a (Cha), transparency (SD), and trophic level index (TLI).

To identify the environmental factors that have mostly affected the phytoplankton communities, correlation analyses were performed between the units contributing to differences in the community structure and environmental variables (Figure 5, Figure 6 and Figure 7, Supplemental Table S3). During the dry season, the COD is significantly and positively correlated with Bacillariophyceae and Cyanobacteria, while in the wet season, it is significantly and positively correlated with Cyanobacteria and Cryptophyta. Conversely, the SD is significantly and negatively correlated with Cyanobacteria and Cryptophyceae during the dry season, and with Cyanobacteria and Bacillariophyceae during the wet season. Additionally, the pH is significantly and positively correlated with Cyanobacteria and Euglenophyta in the dry season, and with Cyanobacteria and Cryptophyceaein the wet season. The TN is significantly and positively correlated with Bacillariophyceae during the dry season, and with Bacillariophyceae and Cyanobacteria during the wet season. The Chla and TLI are closely associated with most algae, except for Dinoflagellata and Chrysophyceae, during the dry season, and with Dinoflagellata, Chrysophyceae, and Euglenophyta in the wet season.

Group A has significant positive correlations between the COD and Bacillariophyceae, Cyanobacteria, and Chlorophyta. Conversely, the Chla and TLI have significant positive correlations with Bacillariophyceae, Cyanobacteria, Chlorophyta, and Cryptophyta. The TN has a significant negative correlation with Dinoflagellata, while SD has a significant negative correlation with Chlorophyta and Cryptophyta. In Group B, Tur is significantly and positively correlated with Cyanobacteria, whereas SD exhibits a significant negative correlation, and TN and TLI have significant negative correlations with Chrysophyceae. In Group C, both TN and TP have significant positive correlations with Bacillariophyceae.

A variation partition analysis (VPA) revealed that COD, pH, DO, and TN are the environmental variables that most affect the phytoplankton community structure (Figure 7).

### 2.4. Co-Occurrence Patterns of Phytoplankton Communities

**Figure 8:**
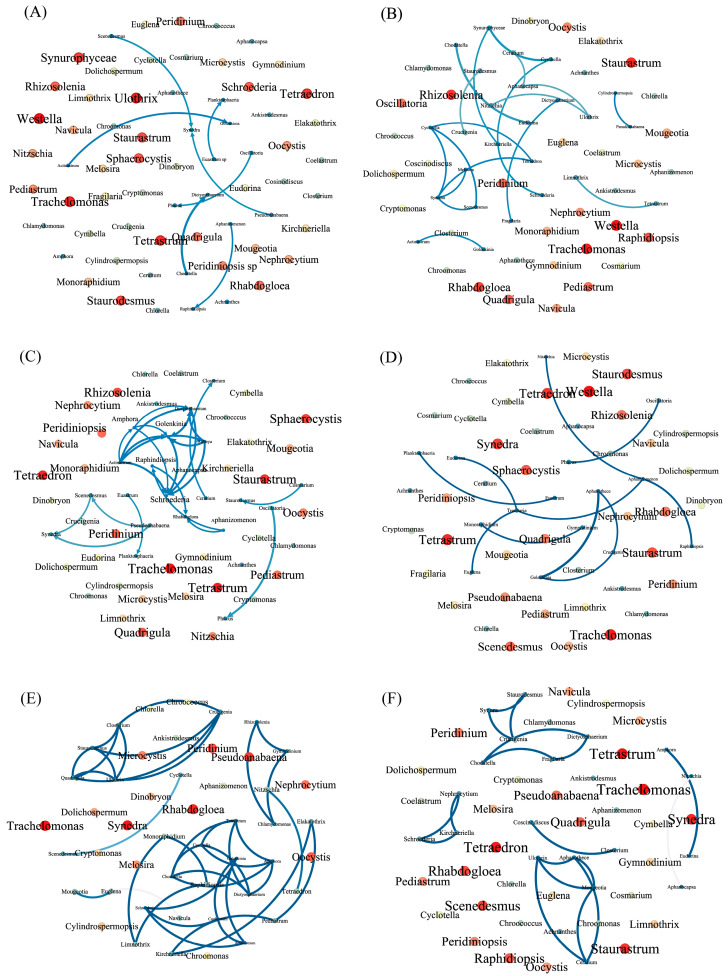
Co-occurrence network analysis. Dot size correlates with phytoplankton density (the larger the dot, the greater the density). The darker the line, the higher the correlation. (**A**–**F**) co-occurrence networks of: (**A**) all data (Group All); (**B**) dry season; (**C**) wet season; (**D**) Group A; (**E**) Group B; (**F**) Group C.

We performed a co-occurrence network analysis, in which the average degree can be used to measure the degree of connectivity or strength of correlation between nodes in a network. The higher the average degree, the denser and stronger the correlation between the network nodes. When using all data in the analysis (Group All), the average degree (0.108) is the lowest. The average wet season value (0.414) is higher than the dry season (0.322) value. Spatially, the average values are 0.812 (Group B), 0.34 (Group C), and 0.193 (Group A) (Figure 8). The species-specific co-occurrence was generally low in Group All. During the dry season, *Synedra* sp. had the highest symbiotic rate compared with the other phytoplankton taxa, but during the wet season, *Golenkinia* sp. had the highest symbiotic rate, which mainly occurred with Cyanobacteria and Chlorophyta. Within Group A, *Aphanizomenon* sp. had the highest symbiotic rate compared with the other species (e.g., Chlorophyta and Bacillariophyceae). In Group B, *Ulothrix* sp., *Quadrigula* sp., *Staurodesmus* sp., *Closterium* sp., and *Amphora* sp. had the highest symbiotic rates, primarily co-occurring among Chlorophyta. In Group C, *Ceratium* sp. and *Mougeotia* sp. had the highest symbiotic rates, being primarily associated with Chlorophyta. Overall, the topological characteristics reveal that the phytoplankton community network is more complex and compact in the wet season and in Group B, but simpler in the dry season and in Group A.

## 3. Discussion

### 3.1. Spatiotemporal Characteristics of Phytoplankton Communities

We reported that the number of phytoplankton species in Shenzhen drinking water reservoirs were similar between the dry and wet seasons, and that they comprised approximately 60 genera in total (Figure S1). During the dry season, the numbers of genera were similar between Groups A and C, but lower in Group B. During the wet season, Group A had more genera than the other groups (Figure S2). These differences may be due to the different water quality statuses in various geographic locations.

As shown in Figure S1, Cyanobacteria dominate the drinking water reservoirs, which is consistent with Wang’s survey results in 2000 [12], indicating a little overall temporal change in the composition of phytoplankton in the seven units. However, in the survey results conducted by Lei et al. in 2011, *Cylindrosporopsis* sp. has the advantage of replacing local *Pseudoanabaena* sp. and *Microcystis* sp. in reservoirs in Guangdong Province [12,13,14,15] (Table 1). For instance, in 2013, the dominant species in the TG Reservoir were *Lyngbya* sp. and *Pseudanabaena* sp. [16], while in the wet season, and even during the cooler dry season, we report that the dominant species is *Cylindrospermopsis* sp., indicating a gradual adaptation of *Cylindrospermopsis* sp. To low temperatures, where it can thrive and flourish [17]. The ability of *Cylindrospermopsis* sp. to produce the cyanotoxin cylindrospermopsin posed a significant challenge to drinking water safety and public health [18], so this phenomenon must be monitored.

Group C had the highest Chao1 diversity index, while Groups A and B had similar values. Because the Pielou’s diversity index was closer to 1 in Group A than in the other groups, Group A had a lower species diversity and the highest homogeneity. Conversely, Group C had the highest species diversity but the lowest evenness; Group B had intermediate values. This disparity may be because of the industrial differences in Shenzhen’s administrative districts (Figure 3). Group C reservoirs occurred mainly in the districts of Bao’an, Guangming, and Longhua, where industry mainly involves manufacturing. Group B’s reservoirs are concentrated in the districts of Futian, Nanshan, and LuoHu, where industry mainly involves finance, science, and technology innovation. Group A reservoirs in the districts of Dapeng, Longgang, Pingshan, and Yantian have low population densities [19]. Manufacturing industries increase N and P discharge, affecting the phytoplankton community structure [20,21].

Whereas the alpha diversity reveals spatial differences in phytoplankton communities, the beta diversity reveals significant temporal and spatial impacts on phytoplankton community structures. This suggests that shifts in the community species composition did not significantly affect the species diversity. These results may be because of alternative species or shifts in the relative abundance of dominant species within communities. When assessing the reservoir aquatic ecology in the region, the beta diversity may be more responsive to the impacts of environmental factors on ecological communities [22].

### 3.2. Relationships between Phytoplankton Members

In the co-occurrence network, positive and negative correlations represent reciprocal and competitive relationships between connected species, respectively [23]. We report the main positive correlations between phytoplankton in Shenzhen drinking water source reservoirs (Figure 8). The water temperatures in these reservoirs are suitable for algal growth, and the waters are rich in bioavailable nutrients [24]. Cyanobacterial blooms also saturate the ecological space, and other phytoplankton respond to cyanobacterial blooms, with positive correlations between phytoplankton community species often dominating. Positive correlations representing reciprocal relationships thereby reduce the direct competition between species and maintain or improve community diversity and stability through processes such as synergism and ecological niche complementarity. This suggests that co-operation between phytoplankton (the phenomenon of interdependence and mutually beneficial symbiosis) can increase the adaptability of communities to local environmental changes and maintain phytoplankton network stability [25].

The network connections and correlations during the dry season and in Group A were sparser and weaker, respectively, probably because the water temperatures were cooler, there were less nutrients, and fewer phytoplankton species and densities were recorded from individual reservoirs. The Group C reservoirs had very high Cyanobacteria densities, loose phytoplankton network connections, and weak correlations, and the Group B reservoirs had lower Cyanobacteria densities with tighter phytoplankton network connections and strong correlations, indicating that the plankton stability was lower in the reservoirs with a high degree of bloom than those with a low degree of bloom, similar to results of [26]. This also suggests that the structure and function of phytoplankton communities will be more seriously damaged when Cyanobacteria dominate [27]. Because the structural characteristics of phytoplankton communities were also more pronounced following spatial and temporal grouping, this suggests that (even at the relatively small scale of Shenzhen City), to accurately reflect the structural characteristics of phytoplankton communities in specific habitats, more detailed grouping is required.

### 3.3. Environmental Factors Affecting Phytoplankton Community Structure

Various statistical methods (e.g., Mantel test and correlation and VPA analyses) were performed to identify the main environmental attributes (COD, WT, Tur, DO, pH, TN, and TP) affecting the phytoplankton community structure (Figure 5, Figure 6 and Figure 7).

The COD is a main driver of cyanobacterial blooms [28], and we report that it must be higher during the dry season than the wet season for Group C to have higher COD levels than Group B, and for Group B to have higher levels than Group A. These findings align with the relative abundances of Cyanobacteria. Additionally, a significant positive correlation between the COD and Cyanobacteria was identified. Hence, the COD can be used to predict cyanobacterial bloom occurrences in South China’s reservoirs.

The WT directly affects phytoplankton growth; it can change the distributions of nutrients and DO in the reservoir and affect phytoplankton photosynthesis and reproduction by influencing enzyme activities [29]. Temporal variation in the WT may affect phytoplankton community evolution. The WT differed significantly between the dry (range of 20–25 °C) and wet (range of 25–30 °C) seasons. A higher WT favors rapid Cyanobacteria reproduction (e.g., *Cylindrospermopsis* sp. and *Microcystis* sp.), but correlates negatively with algal growth and has lower optimum growth temperatures (e.g., Chrysophyceae and Cryptophyta). Consequently, Cyanobacteria were proportionally more abundant during the wet season than other algae groups.

Tur is closely related to SD, which affects phytoplankton photosynthesis. Suspended particulate matter such as sediment adsorbs and desorbs couples with nutrients such as nitrogen and phosphorus through surface interactions, thereby affecting the distribution and transformation of nutrients in water bodies and ultimately affecting the water quality and living environment of phytoplankton [30]. Phytoplankton in water with higher Tur values may be nutrient-limited, thus affecting community composition [30]. The Tur in the Group A reservoirs was lower than in Groups B and C, and the SD of the Group A reservoirs was higher. A correlation analysis revealed that Tur and SD are significantly related to Cyanobacteria and Cryptophyta, which is consistent with Nunes’s survey results that a higher Tur increased the relative abundance of Phagotrophic algae [31].

DO is intrinsically linked to phytoplankton community characteristics because phytoplankton need oxygen to respire, while simultaneously releasing it through photosynthesis. Consequently, variations in DO have profound impacts on the concentrations of other limiting nutrients, including the available iron (Fe), thereby influencing the phytoplankton community composition [32]. It is important to remark that Fe is a redox-sensitive element, and dissolved Fe concentrations mostly depend on the gradients of oxygen and pH condition developing during stratification. Actually, under oxygen-rich conditions and alkaline pH values such as those occurring in the epilimnion of water bodies, highly bioavailable Fe^2+^ is quickly transformed in insoluble Fe^3+^ and made unavailable to phytoplankton [33]. We report no significant difference in the DO concentrations between the dry and wet seasons, but a significant difference in the DO concentrations between Groups A and C. There was also a positive and significant correlation between the DO and density of Bacillariophyceae, Chlorophyta, and Cryptophyta, which is consistent with the results of [34].

Nutrient concentrations (e.g., TN and TP) can alter the trophic state of aquatic environments and affect the phytoplankton community structure [35]. Cyanobacteria growth is closely related to the pH, with higher pH levels favoring growth, and cyanobacterial blooms contributing to higher pH [36].

## 4. Materials and Methods

### 4.1. Phytoplankton Community Alpha and Beta Diversities

**Figure 9:**
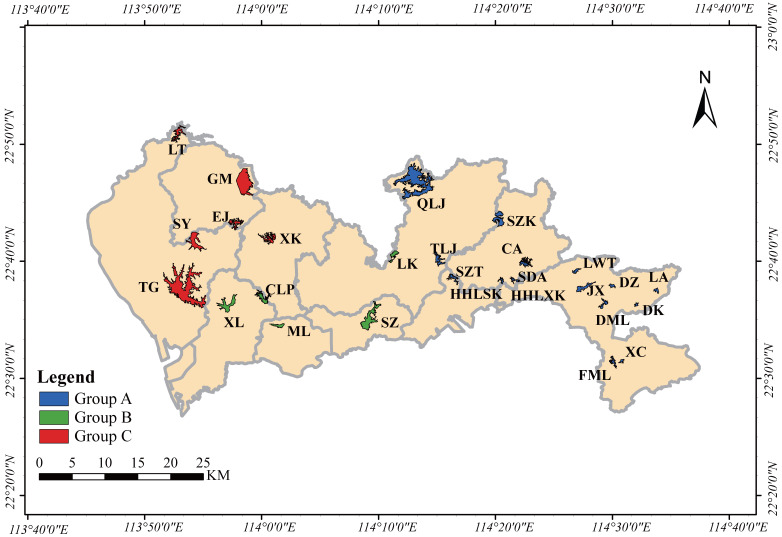
Schematic diagram and grouping of 27 drinking water reservoirs in Shenzhen City. LT: Loutian; GM: Gongming; SY: Shiyan; EJ: Ejing; XK: Xikeng; TG: Teigang; XL: Xili; CLP: Changlingpi; ML: Meiling; SZ: Shenzhen; LK: Longkou; QLJ: Qinglinjing; TLJ: Tongloujing; SZT: Shanzhoutian; SDA: Shangdongao; HHLSK: Honghualingshangku; HHLXK: Honghualingxiaku; LWT: Luowutian; JX: Jingxin; DZ: Dongzi; LA: Lingao; DML: Damali; DK: Dakeng; XC: Xiangche; FML: Fengmulang; CA, Chiao; SZK, Songzikeng.

In this study, 27 drinking water reservoirs were selected for research in December 2021 (dry season) and June 2022 (wet season) in Shenzhen, China (Figure 9). The 27 reservoirs were divided into three groups according to the water supply pipeline and geographical distribution: Group A had 16 reservoirs such as Chiao Reservoir, Dakeng Reservoir, and Damali Reservoir; Group B had five reservoirs such as Changlingpi Reservoir, Longkou Reservoir, and Meilin Reservoir; and Group C had six reservoirs such as Ejing Reservoir, Gomgming Reservoir, and Luotian Reservoir. For each reservoir, we selected the middle of the reservoir as a sampling point to collect phytoplankton.

Phytoplankton samples were quantitatively sampled at different sampling levels according to the water depth of the survey site. The water depth was measured before sampling. When the water depth was less than 5 m or mixed evenly, no stratified sampling was performed, and only water samples at 0.5 m below the water surface were collected. When the water depth was 5–10 m, the water was collected at 0.5 m below the water surface and at the bottom of the transparent layer (the depth was three times the transparency); when the water depth was greater than 10 m, it was collected at 0.5 m below the water surface, half of the transparent layer, and the bottom of the transparent layer. When the water depth was greater than 5 m, if the difference in species and abundance of each layer in the layer was small, the number of layers could be reduced as appropriate.

Stratified water samples of 1 L were collected from each sampling layer using a water sampler, following the order from shallow to deep. The samples collected at each level were poured into a clean bucket prepared beforehand, thoroughly mixed, and 1 L of water sample was transferred into the sample bottle. The sampling volume could be increased as appropriate for the water body in the oligotrophic state. Water samples for phytoplankton detection were fixed by adding 1.5% Ruggier’s solution, recording the information and labeling the sample points, and transported to the laboratory for the next step of quantitative analysis by keeping the samples at a low temperature and protected from light.

### 4.2. Quantitative Identification of Phytoplankton Samples

We collected water samples and brought them back to the laboratory to identify sedimentation, concentration, and constant volume. First, we tested the transparency of the water sample and determined the constant volume based on the relationship between transparency and algae density. Then, we further adjusted and determined the concentration or dilution ratio according to the actual situation of microscopic counting. Finally, we added 0.1 mL of the concentrated water sample to the counting box, which contained about 500~10,000 phytoplankton cells. We used a 1 L volume cylindrical liquid separation funnel to statically precipitate the Lugol’s iodine reagent-fixed water sample for at least 48 h, and gradually sucked away the supernatant through a siphon until the volume of the water sample was concentrated to less than 30 mL of the constant volume, and then transferred it to the sample bottle with the piston of the rotary bottle. Thereafter, the phytoplankton were identified and counted under light microscopy according to the authoritative identification book [37].

We should identify the main species of phytoplankton as much as possible, especially those with indicative significance for the classification of trophic types, and identify at least the genus level. During the identification, we fully shook the concentrated phytoplankton samples and took 0.1 mL into the phytoplankton counting box for identification and counting. We counted 300 cells under a 10 × 40 microscope and identified them to the lowest classification level (genus or species).

We converted the plankton counting results into the density of plankton per liter of water according to the following formula:

$$N=\frac{A}{A_{C}}\times \frac{V_{w}}{V}\times n$$

where N is the number of phytoplankton per liter of water (ind./L); A is the area of the counting box (mm^2^); A_c_ is the counting area (mm^2^), which is the visual field area × the number of visual fields; V_w_ is the volume of 1 L water sample after sedimentation and concentration (mL); V is the volume of the counting box (mL); and n is the number of individuals or cells of phytoplankton obtained by counting.

The standing crop of phytoplankton refers to the number of phytoplankton present in a unit volume of water at a certain time. The unit expressed by number is called quantity, and the unit expressed by mass is called biomass. Because the individual size of different species of plankton varies greatly, it is not accurate enough to use the number of individuals or cells. Therefore, the weight of phytoplankton per unit volume of water is commonly used as a quantitative unit, that is, biomass (wet mass).

### 4.3. Measurement of Environmental Factors

The water quality survey factors at each sampling point included 10 indicators, such as water temperature (WT), pondus hydrogenii (pH), dissolved oxygen (DO), turbidity (Tur), ammonia nitrogen (NH_4_^+^–N), total phosphorus (TP), chemical oxygen demand (COD), total nitrogen (TN), chlorophyll a (Cha), and transparency (SD). The water temperature, pH, and dissolved oxygen were measured using a portable water quality parameter instrument (HACH H40D). The turbidity was measured using a suspended matter turbidity detector (LoH and LH-XZ03). The transparency was measured using a Secchi disk. The physical and chemical indexes of the collected 5 L water samples were measured within 24 h after being transported back to the laboratory. The chemical oxygen demand, chlorophyll a, ammonia nitrogen, total phosphorus, and total nitrogen were determined using the national standard method.

According to the Technical Provisions on Evaluation Methods and Grading of Eutrophication in Lakes (Reservoirs) (China Environmental Monitoring General Station), a comprehensive trophic level index (TLI) was calculated through five items: TP, TN, Chla, COD, and SD.

### 4.4. Phytoplankton Community Alpha and Beta Diversities

Phytoplankton density data were used for data analysis. Species with abundance greater than 15% were defined as dominant species in phytoplankton community composition analysis. The “vegan” package was used to perform alpha diversity indexes (Shannon–Wiener, Simpson, Chao1, Pielou, and Margalef diversity indexes), non-metric multidimensional scaling (NMDS), Mantel analysis, and similarity analysis (ANOSIM). T test was used to test the significance of alpha diversity indexes. Venn plots were performed using the “Venn Diagram” package. Co-occurrence patterns were constructed based on Spearman rank correlation coefficients. Co-occurrence events with a correlation coefficient (|R| > 0.9, *p* < 0.05) were considered statistically significant. The co-occurrence network was visualized in Gephi (version 10.1). All the above analyses were performed using R software (version 4.1.3), and the sampling points were plotted in ArcMap (version 10.1). The figures were revised using Adobe Illustrator (version 2022).

## 5. Conclusions

We report phytoplankton communities in drinking water reservoirs in Shenzhen during the dry and wet seasons. Cyanobacteria dominate in these reservoirs. But the diversity, structure, and taxonomic composition of these communities showed temporal and spatial variations. The phytoplankton diversity was higher during the dry season, while the planktonic plant diversity decreased from the east to west. A co-occurrence network analysis revealed that reservoirs with moderate levels of eutrophication exhibit more dense nodes and stronger correlations compared to those with low or high levels of eutrophication. The COD, WT, pH, and TN were found to be the key factors influencing the phytoplankton community characteristics.

Our multidimensional understanding of the structural characteristics of phytoplankton communities in drinking water reservoirs in Shenzhen provides insights into the maintenance mechanisms of phytoplankton communities. These results also provide a reference for future research on the structural diversity of phytoplankton communities in South China.

## Figures and Tables

**Table 1:** The dominant species of phytoplankton in the drinking water source reservoirs of Shenzhen. Relative abundance values in brackets.

Reservoir	Dry Season	Wet Season
CA	*Microcystis* sp. (0.35), *Cylindrospermopsis* sp. (0.22), *Chlorella* sp. (0.22)	*Cyclotella* sp. (0.36), *Eudorina* sp. (0.21)
DK	*Pseudoanabaena* sp. (0.59), *Chlorella* sp. (0.19)	*Microcystis* sp. (0.25)
DML	*Cryptomonas* sp. (0.38), *Staurastrum* sp. (0.19)	*Staurodesmus* sp. (0.19), *Staurastrum* sp. (0.17)
DZ	*Ankistrodesmus* sp. (0.57)	*Scenedesmus* sp. (0.33), *Cyclotella* sp. (0.25), *Cryptomonas* sp. (0.25), *Cylindrospermopsis* sp. (0.17)
FML	*Coelastrum* sp. (0.35), *Westella* sp. (0.3)	*Coelastrum* sp. (0.69)
HHLSK	*Microcystis* sp. (0.625)	*Oscillatoria* sp. (0.82)
HHLXK	*Scenedesmus* sp. (0.31), *Chlorella* sp. (0.23), *Dolichospermum* sp. (0.15)	*Cyclotella* sp. (0.45)
JX	*Rhizosolenia* sp. (0.28), *Chlorella* sp. (0.17)	*Dolichospermum* sp. (0.44)
LA	*Nephrocytium* sp. (0.33)	*Coelastrum* sp. (0.38), *Cylindrospermopsis* sp. (0.19)
LWT	*Crucigenia* sp. (0.17)	*Nephrocytium* sp. (0.43)
QLJ	*Rhizosolenia* sp. (0.19)	*Limnothrix* sp. (0.26), *Euastrum* sp. (0.26)
SDA	*Oscillatoria* sp. (0.43), *Coelastrum* sp. (0.35), *Coelastrum* sp. (0.35)	*Cyclotella* sp. (0.64), *Melosira* sp. (0.16)
SZK	*Pseudoanabaena* sp. (0.5), *Cylindrospermopsis* sp. (0.17)	*Pseudoanabaena* sp. (0.35), *Microcystis* sp. (0.24), *Limnothrix* sp. (0.29)
SZT	*Chlorella* sp. (0.21), *Dinobryon* sp. (0.21)	*Aphanocapsa* sp. (0.54)
TLJ	*Elakatothrix* sp. (0.27), *Ankistrodesmus* sp. (0.2), *Chlorella* sp. (0.2)	*Cyclotella* sp. (0.72)
XC	*Chlorella* sp. (0.43)	*Coelastrum* sp. (0.72)
CLP	*Microcystis* sp. (0.95)	*Microcystis* sp. (0.99)
LK	*Cryptomonas* sp. (0.22), *Crucigenia* sp. (0.20)	*Pseudoanabaena* sp. (0.5), Limnothrix sp. (0.20)
ML	*Peridinium* sp. (0.21), *Scenedesmus* sp. (0.18)	*Nephrocytium* sp. (0.59)
SZ	*Aphanizomenon* sp. (0.21), *Mougeotia* sp. (0.21)	*Cyclotella* sp. (0.29)
XL	*Melosira* sp. (0.75)	*Dolichospermum* sp. (0.22), *Pseudoanabaena* sp. (0.21)
EJ	*Pseudoanabaena* sp. (0.47)	*Pseudoanabaena* sp. (0.62)
GM	*Pseudoanabaena* sp. (0.83)	*Pseudoanabaena* sp. (0.49), *Limnothrix* sp. (0.21)
LT	*Cryptomonas* sp. (0.22), *Crucigenia* sp. (0.20)	*Limnothrix* sp. (0.41), *Pseudoanabaena* sp. (0.34)
SY	*Pseudoanabaena* sp. (0.34)	*Pseudoanabaena* sp. (0.30), *Aphanizomenon* sp. (0.27), *Microcystis* sp. (0.21)
TG	*Cylindrospermopsis* sp. (0.17), *Dolichospermum* sp. (0.17)	*Pseudoanabaena* sp. (0.51), *Cylindrospermopsis* sp. (0.37)
XK	*Melosira* sp. (0.45), *Dolichospermum* sp. (0.21), *Cyclotella* sp. (0.16)	*Pseudoanabaena* sp. (0.64)

## Data Availability

Data are available on request to the authors.

## Supplementary Materials

The following supporting information can be downloaded at https://www.mdpi.com/article/10.3390/plants12233933/s1, Figure S1: Phytoplankton situation; Figure S2: Venn diagram; Figure S3: α diversity analysis results of 27 reservoirs; Table S1: Phytoplankton composition and density by reservoir; Table S2: Reservoir groupings and abbreviations; Table S3: Percentage similarity of phytoplankton (SIMPER) analysis of drinking water source reservoirs in Shenzhen (top 20); Table S4: *p*-values and R-values for intergroup correlation analysis.
