# Supplementary material for: Spatial and Temporal Characteristics of Phytoplankton Communities in Drinking Water Source Reservoirs in Shenzhen, China
Source: Plants (Basel). 2023 Nov 22;12(23):3933. doi: 10.3390/plants12233933 (PMC10707980; doi:10.3390/plants12233933)
Supplement: Supplementary file 1 [file plants-12-03933-s001.zip › Supplemantary files.pdf]

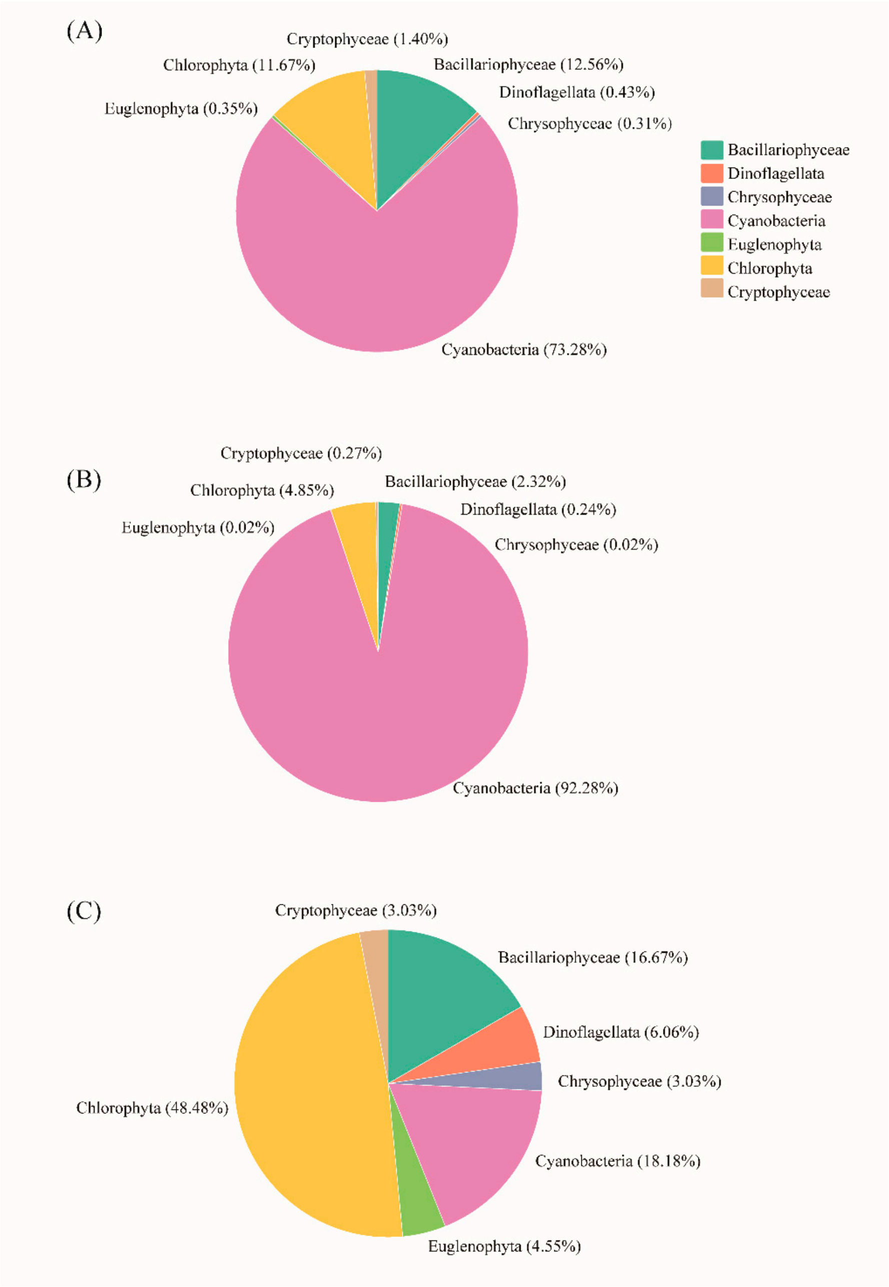

Figure S1. Phytoplankton situation. (A) Phytoplankton density during the dry season; (B) Phytoplankton density during the wet season; (C)Number of genera of species in 7 classification units.

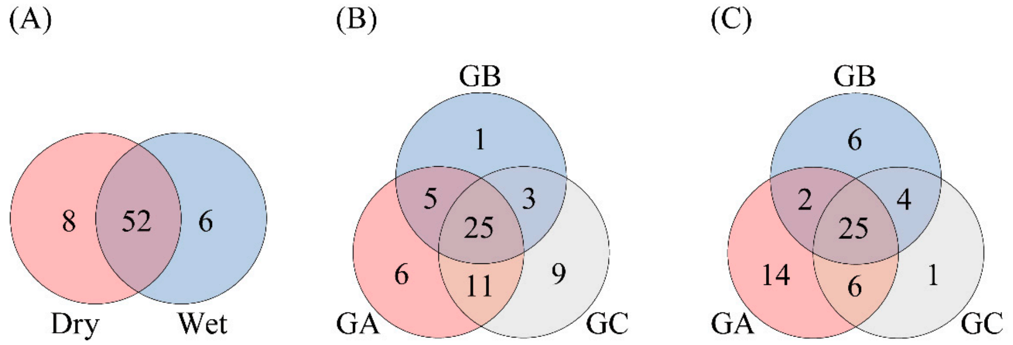

Figure S2. Venn diagram. (A) Venn diagram showing phytoplankton distribution during dry and wet season. Dry represents dry season, Wet represents wet season; (B) Venn diagram showing the distribution of phytoplankton at different group in the dry season. GA, Group A; GB, Group B; GC, Group C; GD, Group D; (C) Venn diagram showing the distribution of phytoplankton at different group in the Wet season. GA, Group A; GB, Group B; GC, Group C; GD, Group D.

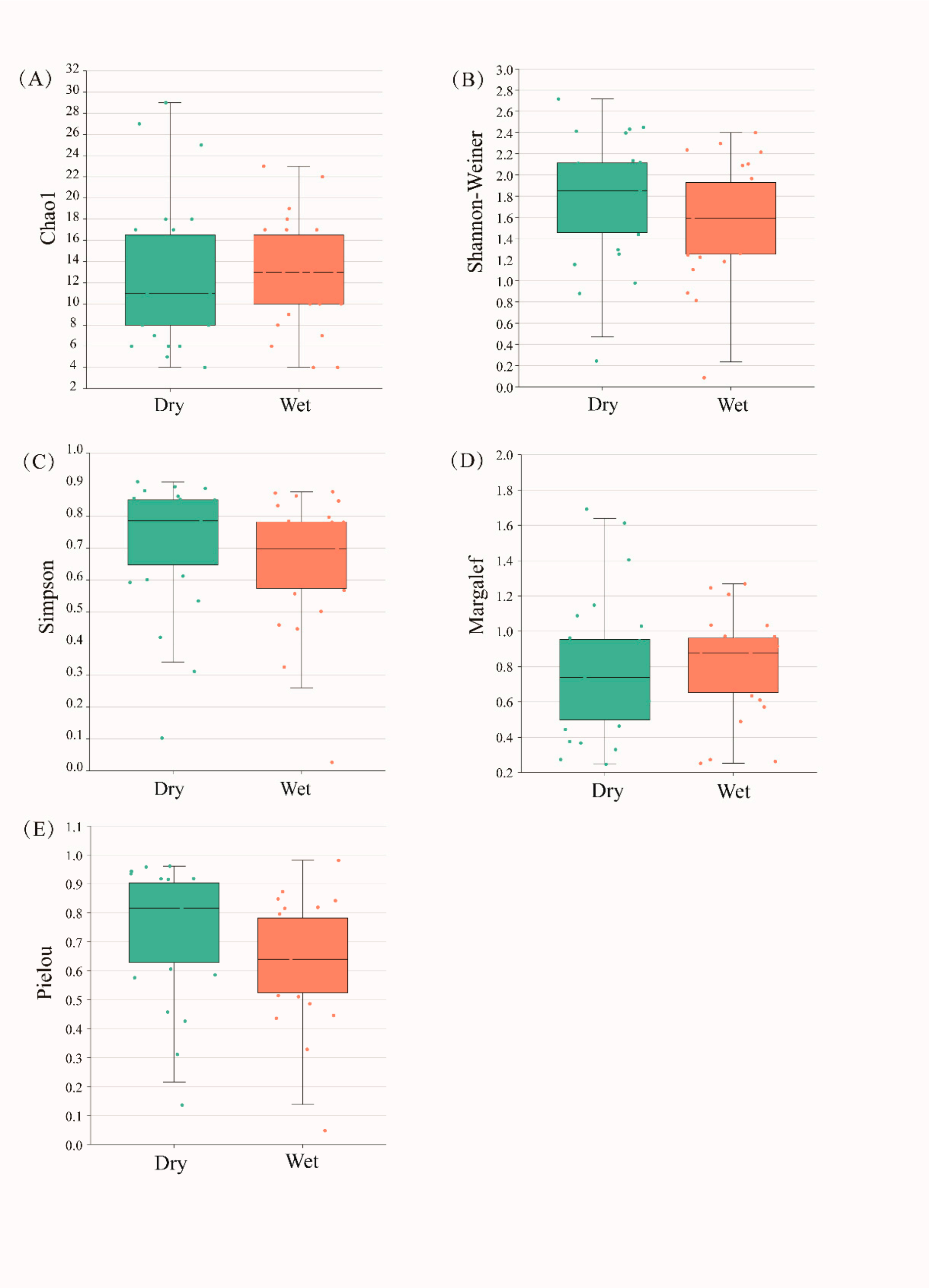

Figure S3. α diversity analysis results of 27 reservoirs. Dry represents dry season, Wet represents wet season. (A) Chao1 index; (B) Shannon-Wiener index; (C) Simpson index; (D) Margalef index; (J)Pielou index.

Table S2. Reservoir groupings and abbreviations

Group	Reservoirs	Abbreviated name
A	Dakeng	DK
	Damali	DML
	Dongzi	DZ
	Fengmulang	FML
	Jingxin	JX
	Ling'ao	LA
	Luowutian	LWT
	Xiangche	XC
	Qinginjing	QLJ
	Tongluojing	TLJ
	Chiao	CA
	Honghualingshangku	HHLSK
	Honghualingxiaku	HHLXK
	Shangdongao	SDA
	Songzikeng	SZK
	Sanzhoutian	SZT
B	Meilin	ML
	Longkou	LK
	Shenzhen	SZ
	Changlingpi	CLP
	Xili	XL
C	Luotian	LT
	Shiyan	SY
	Teigang	TG
	Ejing	EJ
	Gongming	GM
	Xikeng	XK

Table S3. Percentage similarity of phytoplankton (SIMPER) analysis of drinking water source reservoirs in Shenzhen(top 20).

Genus	Average	SD	Ratio	Cumsum
Pseudoanabaena sp.	0.19084	0.22683	0.841	0.214
Microcystis sp.	0.11071	0.24517	0.452	0.339
Melosira sp.	0.05271	0.12443	0.424	0.398
Cylindrospermopsis sp.	0.04981	0.0748	0.666	0.454
Coelastrum sp.	0.04153	0.1138	0.365	0.501
Limnothrix sp.	0.03736	0.08582	0.435	0.543
Scenedesmus.sp.	0.0344	0.03917	0.878	0.581
Cyclotella sp.	0.03028	0.05029	0.602	0.616
Oscillatoria sp.	0.02382	0.10644	0.224	0.642
Dolichospermum sp.	0.02344	0.06189	0.379	0.669
Nephrocytium sp.	0.02331	0.07467	0.312	0.695
Chlorella sp.	0.02241	0.03706	0.605	0.72
Aphanizomenon sp.	0.02206	0.05117	0.431	0.745
Cryptomonas sp.	0.01833	0.04222	0.434	0.765
Ankistrodesmus sp.	0.0148	0.0294	0.503	0.782
Peridinium sp.	0.01192	0.02375	0.502	0.795
Staurastrum sp.	0.01088	0.02942	0.37	0.808
Rhizosolenia sp.	0.01079	0.03208	0.336	0.82
Crucigenia sp.	0.00937	0.03257	0.288	0.83

Table S4 P-values and R-values for intergroup correlation analysis.

Dry season														
	pValue							rValue						
	Bacillariophyta	Pyrrophyta	Chrysophyta	Cyanophyta	Euglenophyta	Chlorophyta	Cryptophyta	Bacillariophyta	Pyrrophyta	Chrysophyta	Cyanophyta	Euglenophyta	Chlorophyta	Cryptophyta
WT	0.4579	0.8011	0.1163	0.2366	0.4433	0.4570	0.3079	-0.1491	-0.0509	-0.3094	0.2357	0.1539	-0.1494	-0.2038
pH	0.0138	0.2351	0.1652	0.0068	0.0056	0.1234	0.4141	0.4682	-0.2364	-0.2749	0.5084	0.5183	0.3038	0.1639
DO	0.3882	0.6991	0.2295	0.9710	0.2905	0.5790	0.5127	0.1730	-0.0780	-0.2392	-0.0073	0.2111	0.1117	0.1317
TUR	0.7289	0.8944	0.1202	0.1270	0.1699	0.7994	0.1521	0.0699	0.0268	-0.3063	0.3011	0.2720	0.0513	0.2833
TRA	0.0065	0.3910	0.0261	0.0001	0.1591	0.0259	0.1063	-0.5105	0.1720	0.4277	-0.6777	-0.2788	-0.4282	-0.3177
NH_4^+-N	0.2416	0.5073	0.6718	0.1273	0.2668	0.9697	0.6090	0.2332	0.1333	0.0854	0.3008	0.2215	0.0077	0.1030
TN	0.0017	0.0416	0.0269	0.0201	0.0299	0.2333	0.1896	0.5745	-0.3946	-0.4255	0.4449	0.4183	0.2373	0.2604
TP	0.2956	0.1323	0.6817	0.3960	0.4722	0.3975	0.7475	0.2089	-0.2971	0.0827	-0.1702	0.1444	0.1697	0.0650
COD_{Mn}	0.0000	0.6380	0.2731	0.0002	0.0621	0.0096	0.0765	0.7698	-0.0948	-0.2187	0.6623	0.3638	0.4891	0.3466
Chla	0.0000	0.9856	0.1383	0.0000	0.0267	0.0004	0.0359	0.7295	0.0036	-0.2928	0.7269	0.4260	0.6326	0.4054
TLI	0.0000	0.3835	0.1545	0.0002	0.0030	0.0019	0.0391	0.7869	-0.1747	-0.2817	0.6590	0.5487	0.5697	0.3993

Wet season														
	pValue							rValue						
	Bacillariophyta	Pyrrophyta	Chrysophyta	Cyanophyta	Euglenophyta	Chlorophyta	Cryptophyta	Bacillariophyta	Pyrrophyta	Chrysophyta	Cyanophyta	Euglenophyta	Chlorophyta	Cryptophyta
WT	0.7701	0.5876	0.6680	0.4668	0.8838	0.0467	0.2843	0.0590	0.1092	-0.0865	0.1462	0.0295	0.3860	0.2138
pH	0.0279	0.6468	0.1948	0.0004	0.7530	0.0133	0.0004	0.4231	0.0924	-0.2575	0.6307	-0.0635	0.4702	0.6293
DO	0.1989	0.3394	0.1162	0.0209	0.5681	0.0362	0.0434	0.2552	0.1912	-0.3095	0.4421	0.1149	0.4048	0.3915
TUR	0.1817	0.6868	0.0424	0.0381	0.5759	0.0519	0.0295	0.2650	-0.0813	-0.3933	0.4011	0.1127	0.3780	0.4193

TRA	0.0278	0.6370	0.0273	0.0009	0.7944	0.0206	0.0006	-0.4233	0.0951	0.4245	-0.6030	0.0526	-0.4432	-0.6203
NH_4^+-N	0.9865	0.1322	0.8210	0.9754	0.0488	0.8862	0.5118	-0.0034	-0.2972	0.0457	-0.0062	-0.3827	0.0289	-0.1319
TN	0.0014	0.2495	0.2339	0.0034	0.1086	0.1129	0.1146	0.5821	-0.2295	-0.2370	0.5431	-0.3158	0.3122	0.3108
TP	0.0061	0.4151	0.0869	0.0012	0.0992	0.0440	0.1528	0.5143	-0.1635	-0.3357	0.5893	-0.3240	0.3906	0.2829
COD_{Mn}	0.0565	0.2012	0.8191	0.0000	0.5923	0.0066	0.0005	0.3713	0.2540	-0.0462	0.7284	0.1079	0.5094	0.6214
Chla	0.0001	0.5389	0.3161	0.0000	0.6300	0.0004	0.0006	0.6717	0.1237	-0.2005	0.7499	-0.0971	0.6369	0.6205
TLI	0.0126	0.8404	0.1984	0.0001	0.5415	0.0171	0.0106	0.4824	0.0415	-0.2607	0.6981	-0.1254	0.4633	0.4926

Group A														
	pValue							rValue						
	Bacillariophyta	Pyrrophyta	Chrysophyta	Cyanophyta	Euglenophyta	Chlorophyta	Cryptophyta	Bacillariophyta	Pyrrophyta	Chrysophyta	Cyanophyta	Euglenophyta	Chlorophyta	Cryptophyta
WT	0.8249	0.8112	0.0101	0.8277	0.8515	0.4055	0.0135	-0.0407	0.0440	-0.4483	-0.0401	0.0345	-0.1522	-0.4321
pH	0.5304	0.8498	0.5344	0.5298	0.6380	0.6080	0.3314	-0.1151	-0.0348	-0.1140	0.1153	0.0864	0.0942	-0.1774
DO	0.9318	0.3637	0.4759	0.5159	0.5780	0.0353	0.1564	0.0158	0.1661	-0.1307	0.1192	0.1022	0.3734	0.2565
TUR	0.9365	0.6764	0.1472	0.6661	0.4370	0.0618	0.1653	-0.0147	-0.0767	-0.2621	-0.0793	0.1424	0.3339	0.2513
TRA	0.1610	0.6158	0.3687	0.4256	0.8601	0.0047	0.0016	-0.2538	0.0922	0.1644	-0.1459	-0.0324	-0.4873	-0.5351
NH_4^+-N	0.6566	0.3906	0.6230	0.7715	0.3427	0.8313	0.0357	0.0817	-0.1571	0.0903	0.0534	-0.1734	0.0392	0.3726
TN	0.1222	0.0024	0.7349	0.7250	0.3612	0.5389	0.6807	0.2789	-0.5173	-0.0623	0.0647	-0.1669	-0.1128	0.0756
TP	0.7135	0.9934	0.0314	0.3574	0.9396	0.0254	0.0661	0.0675	0.0015	0.3810	0.1682	0.0140	0.3946	0.3288
COD_{Mn}	0.0032	0.2021	0.7316	0.0043	0.5356	0.0030	0.0664	0.5045	0.2316	0.0631	0.4913	0.1137	0.5085	0.3285
Chla	0.0388	0.4752	0.0590	0.0090	0.6048	0.0001	0.0001	0.3670	0.1309	0.3373	0.4541	0.0951	0.6275	0.6501
TLI	0.0430	0.7957	0.1546	0.0308	0.8842	0.0006	0.0009	0.3600	-0.0476	0.2576	0.3824	0.0268	0.5723	0.5574

Group B

	pValue							rValue						
	Bacillariophyta	Pyrrophyta	Chrysophyta	Cyanophyta	Euglenophyta	Chlorophyta	Cryptophyta	Bacillariophyta	Pyrrophyta	Chrysophyta	Cyanophyta	Euglenophyta	Chlorophyta	Cryptophyta
WT	0.3736	0.8809	0.8868	0.1869	0.7353	0.7892	0.4657	-0.3161	0.0546	0.0519	0.4545	-0.1228	-0.0973	0.2614
pH	0.8022	0.2397	0.5816	0.3104	0.7353	0.5784	0.5096	0.0912	-0.4097	-0.1989	0.3576	-0.1228	0.2006	0.2371
DO	0.9601	0.2755	0.6155	0.6515	0.6040	0.4874	0.2505	0.0182	-0.3824	-0.1816	0.1636	0.1875	0.2492	0.4012
TUR	0.6021	0.7068	0.1033	0.0008	0.0818	0.7253	0.3001	-0.1885	-0.1366	-0.5449	0.8788	-0.5754	-0.1277	-0.3647
TRA	0.8277	0.2381	0.1021	0.0072	0.0807	0.9334	0.3482	-0.0793	0.4109	0.5466	-0.7842	0.5771	0.0305	0.3323
NH_4^+-N	0.7840	0.1135	0.6958	0.7715	0.2139	0.7840	0.1054	0.0997	0.5320	0.1419	0.1056	-0.4308	-0.0997	-0.5422
TN	0.1921	0.0518	0.0294	0.7261	0.7353	0.4339	0.8807	0.4499	-0.6282	-0.6833	0.1273	-0.1228	0.2796	-0.0547
TP	0.2658	0.1291	0.1354	0.8272	0.8438	0.2740	0.8862	0.3896	-0.5134	-0.5063	0.0795	0.0718	0.3834	-0.0521
COD_{Mn}	0.1927	0.3706	0.9426	0.0560	0.4088	0.5042	0.6839	-0.4492	0.3180	-0.0263	0.6197	-0.2945	-0.2400	-0.1477
Chla	0.0596	0.4145	0.1054	0.5208	0.0986	0.1223	0.5423	0.6128	-0.2911	-0.5423	0.2310	-0.5512	0.5213	-0.2195
TLI	0.3833	0.0924	0.0294	0.0897	0.5296	0.3639	0.9867	0.3100	-0.5599	-0.6833	0.5636	-0.2263	0.3222	-0.0061

Group C														
	pValue							rValue						
	Bacillariophyta	Pyrrophyta	Chrysophyta	Cyanophyta	Euglenophyta	Chlorophyta	Cryptophyta	Bacillariophyta	Pyrrophyta	Chrysophyta	Cyanophyta	Euglenophyta	Chlorophyta	Cryptophyta
WT	0.6881	0.1982	0.2648	0.0137	0.0529	0.1985	0.8115	0.1296	0.3995	-0.3500	0.6865	-0.5703	0.3993	-0.0772
pH	0.5128	0.0234	0.3339	0.1591	0.3365	0.7954	0.5121	0.2098	0.6454	-0.3057	0.4336	-0.3041	0.0839	0.2102
DO	0.0479	0.0868	0.4954	0.5717	0.4954	0.7456	0.1817	0.5804	0.5148	-0.2184	0.1818	-0.2183	0.1049	0.4133
TUR	0.1063	0.2783	0.8928	0.6331	0.5603	0.9484	0.9483	-0.4895	0.3408	-0.0437	0.1538	0.1872	0.0210	-0.0210
TRA	0.0296	0.3271	0.5851	0.0879	0.5171	0.5324	0.5539	0.6257	-0.3098	0.1756	-0.5132	0.2077	-0.2004	0.1901
NH_4^+-N	0.9044	0.1480	0.2593	0.0549	0.0122	0.8269	0.3292	-0.0389	-0.4442	0.3537	-0.5664	0.6948	-0.0708	-0.3085
TN	0.0071	0.6031	0.4938	0.3346	0.9519	0.6795	0.6391	0.7298	-0.1674	-0.2191	-0.3053	0.0196	-0.1333	0.1511

TP	0.0044	0.2465	0.7850	0.4051	0.2933	0.5376	0.3243	0.7562	-0.3628	-0.0883	-0.2650	-0.3310	0.1979	0.3115
COD_{Mn}	0.7575	0.9454	0.5796	0.2257	0.4475	0.0205	0.8513	0.0998	-0.0222	-0.1781	-0.3780	0.2425	-0.6561	0.0607
Chla	0.8454	0.6683	0.8924	0.2738	0.1003	0.5113	0.4927	0.0632	0.1383	-0.0438	-0.3439	0.4969	-0.2105	0.2197
TLI	0.2356	0.6529	0.3339	0.3423	0.2306	0.5567	0.8627	0.3706	0.1450	-0.3057	-0.3007	0.3743	-0.1888	0.0560
